# Digital Morphometrics of Two North American Grapevines (*Vitis*: Vitaceae) Quantifies Leaf Variation between Species, within Species, and among Individuals

**DOI:** 10.3389/fpls.2017.00373

**Published:** 2017-03-17

**Authors:** Laura L. Klein, Madeleine Caito, Chad Chapnick, Cassandra Kitchen, Regan O’Hanlon, Dan H. Chitwood, Allison J. Miller

**Affiliations:** ^1^Department of Biology, Saint Louis University, St. LouisMO, USA; ^2^Science and Conservation Department, Missouri Botanical Garden, St. LouisMO, USA; ^3^Independent Researcher, St. LouisMO, USA

**Keywords:** digital morphometrics, leaf shape, *Vitis*, generalized Procrustes analysis, elliptical Fourier descriptors, linear discriminant analysis

## Abstract

Recent studies have demonstrated that grapevine (*Vitis* spp.) leaf shape can be quantified using digital approaches which indicate phylogenetic signal in leaf shape, discernible patterns of developmental context within single leaves, and signatures of local environmental conditions. Here, we extend this work by quantifying intra-individual, intraspecific, and interspecific variation in leaf morphology in accessions of North American *Vitis riparia* and *V. rupestris* in a common environment. For each species at least four clonal replicates of multiple genotypes were grown in the Missouri Botanical Garden Kemper Center for Home Gardening. All leaves from a single shoot were harvested and scanned leaf images were used to conduct generalized Procrustes analysis, linear discriminant analysis, and elliptical Fourier analysis. Leaf shapes displayed genotype-specific signatures and species distinctions consistent with taxonomic classifications. Leaf shape variation within genotypes and among clones was the result of pest and pathogen-induced leaf damage that alters leaf morphology. Significant trends in leaf damage caused by disease and infestation were non-random with respect to leaf position on the shoot. Digital morphometrics is a powerful tool for assessing leaf shape variation among species, genotypes, and clones under common conditions and suggests biotic factors such as pests and pathogens as important drivers influencing leaf shape.

## Introduction

The diversity of leaf morphologies reflects the multifaceted interplay of genetics, development, and environment. The genetic basis of leaf morphology is currently understood to be influenced both through variation in gene sequence and expression patterns but much remains unknown (Ichihashi et al., 2014; Chitwood and Sinha, 2016). Developmental biology has made great strides in explaining leaf shape variation, identifying constraints on heteroblasty, cell expansion, polar development, and metabolic pathways (Dkhar and Pareek, 2014; Chitwood and Sinha, 2016). Particularly in the case of angiosperms, these genetic and developmental constraints are intertwined with the life history of the organism. Traits such as plant architecture, venation patterns, or total leaf area represent functional tradeoffs that evolved in response to water distribution, drought and freezing tolerance, transpiration efficiency, light exposure, and many other challenges (Kaplan, 2001; Nicotra et al., 2011).

In addition to genetic and developmental effects, aspects of the abiotic and biotic environments influence leaf shape. For example, environments with greater temperature fluctuations correlate with higher incidences of plasticity in leaf shape (Little et al., 2010), and colder climates are associated with higher incidences of larger-toothed, more highly dissected leaf margins (Boyce, 2008; Royer et al., 2009; Peppe et al., 2011). Biotic influences including leaf-borne pests and pathogens add additional complexity to leaf shape. Fungal and viral infections or insects can affect the health of a plant often through leaf and tissue deformity (e.g., Kadioglu et al., 2012). In response to these infections, a series of physical and biochemical processes within the plant (e.g., stomatal closure, changes in ion concentration, induction of reactive oxygen species, up-regulation of genes, etc.; Boyd et al., 2013) result in the expression of a diseased phenotype. Thus, it is important to account for local environmental conditions when interpreting complex phenotypes.

Within the genus *Vitis*, leaf morphology has proven so informative a trait for cultivated varieties that an entire discipline, ampelography, has been devoted to the description of grape leaves (Rendu, 1854). Ampelography was originally developed to identify leaves of cultivated *V. vinifera* L. varieties. The technique has evolved from manually acquired measurements of veins, sinuses, and teeth (Galet, 1979), to a more precise, digital approach utilizing scanned leaf images and rigorous statistical analyses (Chitwood et al., 2014). Recently, digital morphometrics has been employed to describe leaf shape in hundreds of *V. vinifera* varieties (Chitwood et al., 2014), as well as *V. vinifera* hybrids, and among *Vitis* and *Ampelopsis* species (Chitwood et al., 2016a). This work demonstrated that subtle shape variation is unique to different taxa and developmental stages. These important contributions demonstrate that, under common conditions, genetics, and development interact to influence leaf shape in individual vines.

A persistent question among plant morphologists is the extent to which leaf shape varies within and among genotypes. Because grapevines are easily cloned, it is possible to assess intra- and inter-individual variation in a statistically explicit fashion by examining multiple replicates of the same genotype(s) under common and unique environmental conditions (e.g., Atlan et al., 2015). Phenotypic plasticity research indicates that plants respond to their environments at the sub-genotype level, and that there is variation among genotypes in phenotypic response to light (e.g., a shade leaf may be phenotypically different from a sun leaf on the same plant; De Kroon et al., 2005). In grapevines and many other clonally propagated perennial crops, leaf shape plasticity serves as a proxy indicating the range of variation exhibited by genotypes in response to climate. Through morphology we can observe the range of variation expressed by an individual, and can quantify how traits vary not only in different parts of the same plant, but among individuals, populations, and ultimately species.

In this study, we explored intra-and inter-individual variation in leaf shape in two North American *Vitis* species, *V. riparia* Michx. and *V. rupestris* Scheele. These species are closely related and are differentiated morphologically, genetically, and with respect to the environmental variables characterizing their native ranges (Miller et al., 2013; Callen et al., 2016). We quantified leaf shape in at least four clonal replicates of multiple *V. riparia* and *V. rupestris* genotypes growing in a common garden housed at the Missouri Botanical Garden (MBG; St. Louis, MO). Our goals were to: (1) assess variation in leaf shape among the species *V. riparia* and *V. rupestris*, as well as among genotypes within these species, and among clones within genotypes; (2) investigate effects of naturally occurring pests and pathogens on leaf morphology.

## Materials and Methods

### Assessing Leaf Shape Variation among Species, among Genotypes within Species, and among Clones within Genotypes

#### Study System and Research Vineyards

To investigate differences in leaf shape within and among genotypes, and among species, we selected multiple genotypes of two closely related native North American grapevine species, *V. riparia* and *V. rupestris* (Miller et al., 2013; Wan et al., 2013). These species differ in the climatic variables characterizing their environmental niches, in growth habit, and habitat preference (Callen et al., 2016; Moore and Wen, 2016). Both species can be propagated vegetatively with ease and are commonly used for rootstock breeding.

A research vineyard was established at the Missouri Botanical Garden’s William T. Kemper Center for Home Gardening in 2013 (MBG common garden) using canes (dormant shoot clippings) obtained from accessions housed in the USDA Agricultural Research Service Grape Genetics Research Unit germplasm reserve (USDA-ARS-GGRU; Geneva, NY, USA). The garden plot was open to observation in the public area of the botanical garden grounds, with a center experimental plot and a side experimental plot, divided by a pathway (Supplementary Figure S1). Four genotypes of *Vitis riparia* and five genotypes *V. rupestris* were planted in a randomized design in the MBG common garden, each with at least four clonal replicates per genotype (clones) (**Table 1**). The *V. riparia* and *V. rupestris* genotypes in the MBG common garden represent a subset of the variation preserved at the USDA-ARS-GGRU germplasm.

**Table 1:** Summary of MBG common garden germplasm accessions.

Species	Genotype	No. clones at MBG	Sex	Genotype origin
*Vitis riparia*	588347	7	Female	Illinois
	588354	8	Male	Illinois
	588439	7	Female	Missouri
	588653	7	Female	Iowa
*Vitis rupestris*	588160	7	Female	Illinois or Texas
	588224	4	Female	Arkansas
	588181	5	Male	Missouri
	588188	6	Male	Missouri
	588333	7	Male	Missouri
Total	9	58		

*Genotype represents the USDA-ARS-GGRU germplasm accession from which canes were harvested. Genotype origin is the putative location from where accessions were originally harvested.*

Leaf collections were made June 29, 2014 from single shoots directly spawned from buds on the previous year’s spurs whenever possible. We selected well-established axillary vines in the event primary shoots were previously pruned. Leaf blades were harvested from the shoots and placed into plastic bags with ventilation holes. To preserve the order of developmental leaf stage along a selected shoot, leaves were stacked from youngest (open, fully developed at the tip of the shoot, ∼1 cm in diameter; numbered as one) to oldest leaf at the base of the vine. Leaves were digitally imaged using a Canon CanoScan LiDE 110 color image scanner within 24 h of harvest. Occasionally some leaves were damaged or missing from a shoot as the result of tissue damage from weather or herbivory and were thus excluded in the numbering process. All images are available on the Dryad Digital Repository^1^.

#### Analyses

**FIGURE 1:**
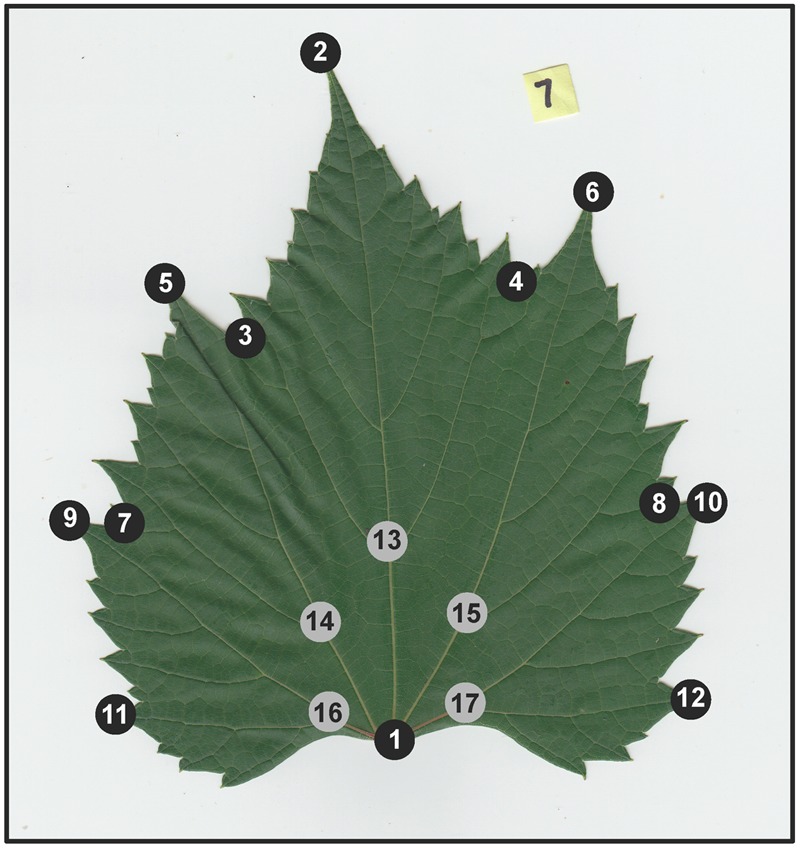
**Example of a scanned *Vitis riparia* leaf, with 17 landmark points applied.** Black dots correspond to 12 “outer landmarks” that capture leaf outline, sinuses, and lobes. Gray dots correspond to five “inner landmarks” that capture aspects vein architecture.

To identify leaf shape variation, we utilized generalized Procrustes analysis (GPA), a method of shape comparison that scales the data equally to eliminate the effects of different-sized objects, resulting in an analysis that examines differences among shapes only (Viscosi and Cardini, 2011). GPA is applied to landmark data that represent homologous points of shape, in this case important grapevine leaf features. Following Chitwood et al. (2014, 2016a) we applied 17 landmark points on each leaf to capture details of the leaf outline such as lobes and sinuses (12 “outer landmarks”) and vein architecture (5 “inner landmarks”) (**Figure 1**). Landmarks were placed on leaf images using the software package ImageJ (Abràmoff et al., 2004). Following landmark dataset assembly, GPA was implemented in R (R Core Team, 2016) using the ‘procGPA’ function in the “shapes” package (Dryden, 2017), generating 34 principal component (PC) scores for each leaf and percent variance explained by each PC. Eigenleaves were visualized using the ‘shapepca function.’ Visualization of average shape outlines extracted from Procrustes coordinates for each genotype were plotted using custom R scripts and in the R package ggplot2 (Wickham, 2009). All code is available on GitHub^2^.

In order to further investigate differences in leaf shape within genotypes among clones, among genotypes, and among species, we performed linear discriminants analysis (LDA) on the landmark data using R. LDA is a statistical classification method consisting of mechanized pattern detection that can be used to distinguish two or more classes of objects in a dataset (e.g., species, genotypes, or disease). Linear discriminants were determined using the ‘lda’ function in the R package MASS (Venables and Ripley, 2002). Those linear discriminants, which are multivariate classifications similar to PCs, are then used to classify the leaves in the data set, blind to their assigned identity, according to class (i.e., species, genotype, or disease) using the ‘predict’ function. The end result is visualized as a table of predicted vs. actual class (i.e., species, genotype, disease) identity.

A second approach using elliptical Fourier descriptors (EFDs) was employed to look at differences in overall leaf shape within and among genotypes and species. Individual scanned leaves were converted to binary images (i.e., black leaf image on a white background) using custom macros in ImageJ for chain coding. Occasionally, some leaves were damaged or diseased, resulting in deformed leaf shapes (see below); these leaves were removed from the EFD dataset. Each binary image was converted into chain code using the program SHAPE v1.3 (Iwata and Ukai, 2002). EFD analysis begins by building chain code along the perimeter of each leaf to create a harmonic series (Chitwood and Sinha, 2016). Chain code contours were converted to normalized EFDs for Fourier analysis. In the R package Momocs (Bonhomme et al., 2014), function ‘nef2coe’ was used to convert normalized EFDs to harmonic coefficients, or ‘coe’ objects. The ‘coe’ objects were analyzed for differences in leaf shape outline using PCA and visualized using the ‘dudi.plot’ function. For each genotype, an average outline shape was calculated using the ‘meanShapes’ function, then visualized using function ‘tps.iso’ in the Momocs R package.

### Signatures of Pest and Pathogen Interaction

The MBG common garden included North American grapevines grown within their natural geographic distribution and were not treated with pesticides or fungicides in the common garden. Consequently, several individuals became infested with phylloxera (*Daktulosphaira vitifoliae* Fitch) and infected with grape fan-leaf virus (GFLV), among potential others. Both are common issues among native North American *Vitis* species and are generally non-fatal, but these pests and pathogens can have significant effects on the morphology of grapevine leaves and are disastrous in the grape industry (Andret-Link et al., 2004; Nabity et al., 2013). During our preliminary analyses, we detected leaf morphologies atypical of *V. riparia* or *V. rupestris*; subsequently, we determined these leaves were infected with phylloxera galls and/or GFLV. While this is an interesting aspect of phenotype, it reduces the accuracy with which we can interpret differences between species and within and among genotypes. Therefore, we removed those individuals expressing the diseased phenotype for examining differences between healthy individuals and performed separate analyses on a dataset including the diseased phenotype.

Two resulting data sets and analyses were designed to assess these aspects of leaf shape: (1) a phenotypically disease-free dataset to address overall differences in leaf shape using GPA, LDA, and EFDs, with individual leaves that expressed the GFLV phenotype as well as any leaves laden with phylloxera galls that severely deformed leaf morphology removed; (2) the total dataset (i.e., including diseased and non-diseased phenotypes) to assess the impact of disease on morphology. Leaves in the total dataset were scored based on the presence of a phylloxera- or GFLV-infected genotype, and correlation tests between shoot position and those leaves expressing the diseased phenotypes were performed using Spearman’s rank correlation rho and visualized with ggplot2 (Wickham, 2009).

## Results

### Assessing Leaf Shape Variation among Species, among Genotypes within Species, and among Clones within Genotypes

**FIGURE 2:**
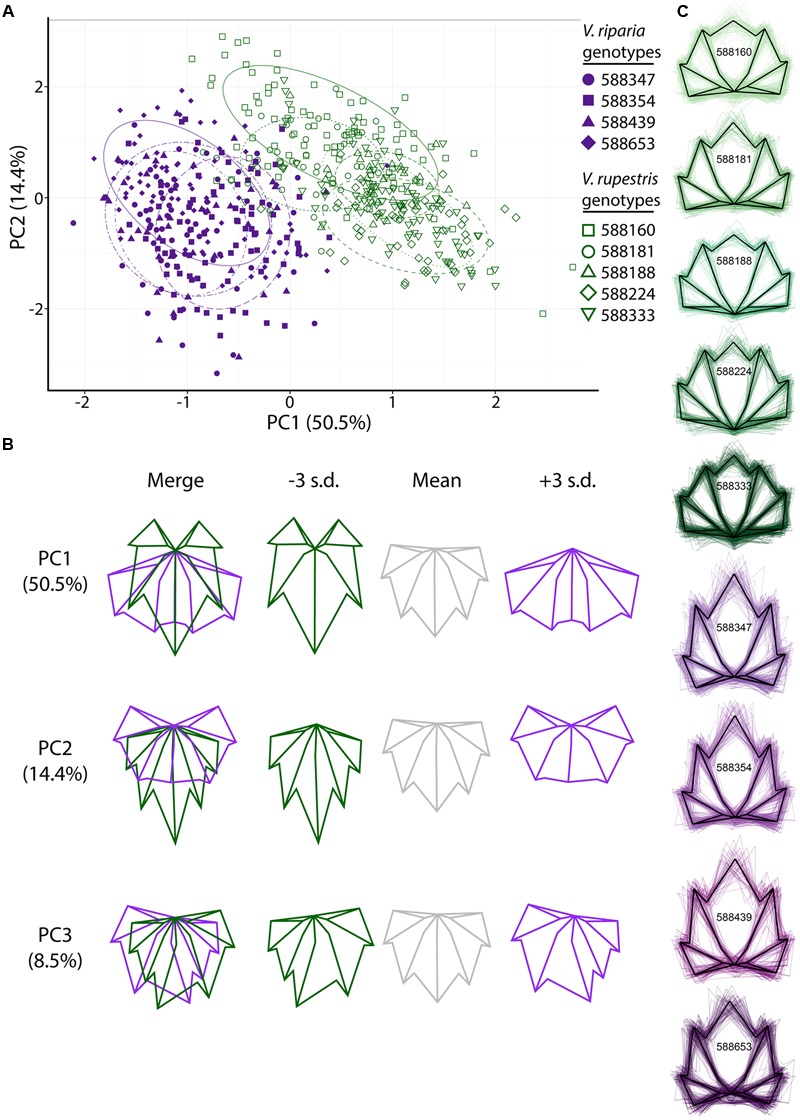
**Generalized Procrustes analysis (GPA) of 17 landmark points applied to leaves harvested from the MBG common garden principal components (PC) morphospace. (A)** PCs 1 and 2 of *Vitis riparia* (purple, filled) and *V. rupestris* (green, open) leaves. Different shapes represent genotypes (see legend). The 95% confidence ellipses drawn around each genotype are designated by different dashed patterns for each genotype. **(B)** ‘Eigenleaves’ display differences among mean leaf morphologies in PCs 1–3 at ±3 SD and percent shape variance for each. **(C)** Black outline represents the average shape outline of each *V. riparia* and *V. rupestris* genotype, with all outlines super imposed beneath in purple and green, respectively.

To assess variation in leaf shape among species, among genotypes within species, and among clones within genotypes, we first looked at leaf shapes of the phenotypically disease-free *V. riparia* and *V. rupestris* leaves in the MBG common garden. GPA of the landmark points demonstrates observable differences between the leaves of *V. riparia* and *V. rupestris*: the first two PCs explain 64.9% of the variance in the data, with discernible clouds representing *V. riparia* (purple) and *V. rupestris* (green; **Figure 2A**). Low PC1 (**Figure 2A** *x*-axis, **Figure 2B** top panel) scores are reflective of longer than wide leaf blades, deeper petiolar sinuses, and major and minor vein axes (i.e., the inner five landmarks) that vary from the branch point of the midvein being the most distal from the petiolar junction to the midvein branch point and the branch point of both major distal veins being nearly equally distal from the petiolar junction. High PC1 scores describe those leaves that are wider than long, with shallower petiolar sinuses, which is representative of *V. rupestris* leaf morphology. Similar to PC1, low PC2 scores are representative of cordate leaves, but the petiolar sinus largely absent, more convex. High PC1 and PC2 scores also detect wider than long leaf blades, but with shallow yet deeper petiolar sinus lobes characterizing high PC2 scores (**Figure 2A** *y*-axis, **Figure 2B** middle panel).

Generalized Procrustes analysis also detected differences among genotypes within species in the MBG common vineyard. Genotypes (**Figure 2A**) are represented as different shapes (i.e., *V. riparia* genotypes are filled shapes, *V. rupestris* are open), and appear to occupy distinct groups within each species. PC3 detects mostly asymmetrical leaf shape variation (**Figure 2B** bottom panel), a relatively common phenomenon in grape leaves (Wolf et al., 1986). **Figure 2C** represents the mean shape of each genotype extracted from Procrustes coordinates (black outline), as well as all leaf shape outlines (colored), demonstrating that within a species, there are subtle variations within genetically distinct individuals.

**FIGURE 3:**
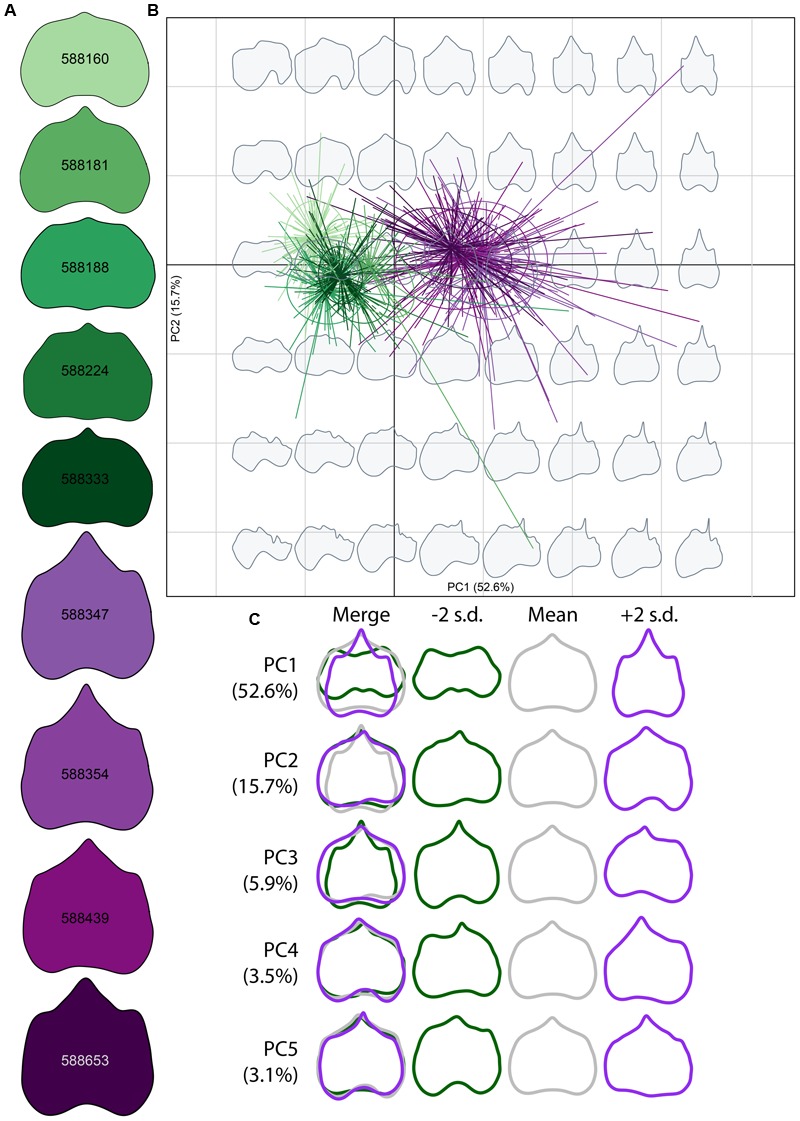
**Morphospace of elliptical Fourier descriptors (EFDs). (A)** Average leaf shape outline for each genotype. Different shades of purple and green correspond to different genotypes. **(B)** PCs 1 and 2 of harmonic coefficients for *V. riparia* (purple) and *V. rupestris* (green). Different shades of purple and green correspond with genotypes in **(A)**. Gray leaf outlines on the background represent shapes drawn on the positions on the factorial map. **(C)** ‘Eigenleaves’ display differences among mean leaf morphologies in PCs 1–5 at ±2 SD and percent shape variance for each, with overlay in the left column.

We used LDA to examine if phenotypically disease-free leaf morphology patterns among species and among genotypes in the MBG common garden vary predictably. Six of 263 *V. riparia* leaves (2%) and three of 315 *V. rupestris* leaves (0.9%) were wrongly classified (**Table 2**; >98% leaves correctly assigned to species). Accuracy decreased when we used LDA to predict genotype for each leaf: 385 of 578 leaves were predicted to be the correct genotype (66%; Supplementary Table S1). Five of the nine genotypes had leaves that were assigned to the wrong species. Two leaves of a single *V. riparia* genotype 588653 clone were incorrectly predicted to be *V. rupestris*. These misclassifications are likely because the genotype tends to be characterized by a shorter midvein, thus appearing more similar to the *V. rupestris* morphology (e.g., **Figures 2C, 3A**). Further, two misclassified leaves from the same individual, among other leaves from this genotype, suggests interclonal variation within this genotype. Out of five clones, two clones of *V. rupestris* genotype 588181 each had one leaf incorrectly predicted to be *V. riparia*. Compared to other *V. rupestris* genotype average shape outlines (**Figures 2C, 3A**), *V. rupestris* genotype 588181 is characterized by a comparatively deeper petiolar sinus, which may have contributed to the incorrect prediction of these leaves. Overall, LDA performed well at identifying leaf shape features at the species level, but accuracy was limited in the classification of leaf shape features at the level of genotype.

**Table 2:** LDA-generated species identity predictions for 263 *V. riparia* leaves and 315 *V. rupestris* leaves.

		Predicted Species Identity	
		*V. riparia*	*V. rupestris*
Species identity	*V. riparia*	257	6
	*V. rupestris*	3	312

*Rows represent species identity according to USDA-ARS-GGRU germplasm accession records, and columns are the predicted species identity based on algorithms the program uses to discern shape features as different categories.*

Elliptical Fourier descriptor analysis, which compares total shape variation through the use of shape outlines (**Figure 3**), largely supports among-species and among-genotype shape variation detected using GPA (**Figure 2**). Low PC1 (**Figure 3B** *x*-axis, **Figure 3C** top panel) values describe those leaf blades that are wider and long, while high PC1 values describe leaf longer than wide blades, for a total of 52.6% of the variation explained in the total dataset. PC2 (**Figure 3B** *y*-axis, **Figure 3C** second panel), describing 15.7% of the variation, captures shallow petiolar sinuses in low PC2 values, verses deeper petiolar sinuses represented by high PC2 values. PCs three through five (**Figure 3C** bottom three panels) detect the asymmetry that characterizes some malformed leaves in the MBG common garden, much like GPA (**Figure 2B**). Asymmetry in leaf shape is common among *Vitis* species (Wolf et al., 1986).

### Signatures of Pest and Pathogen Interaction

**FIGURE 4:**
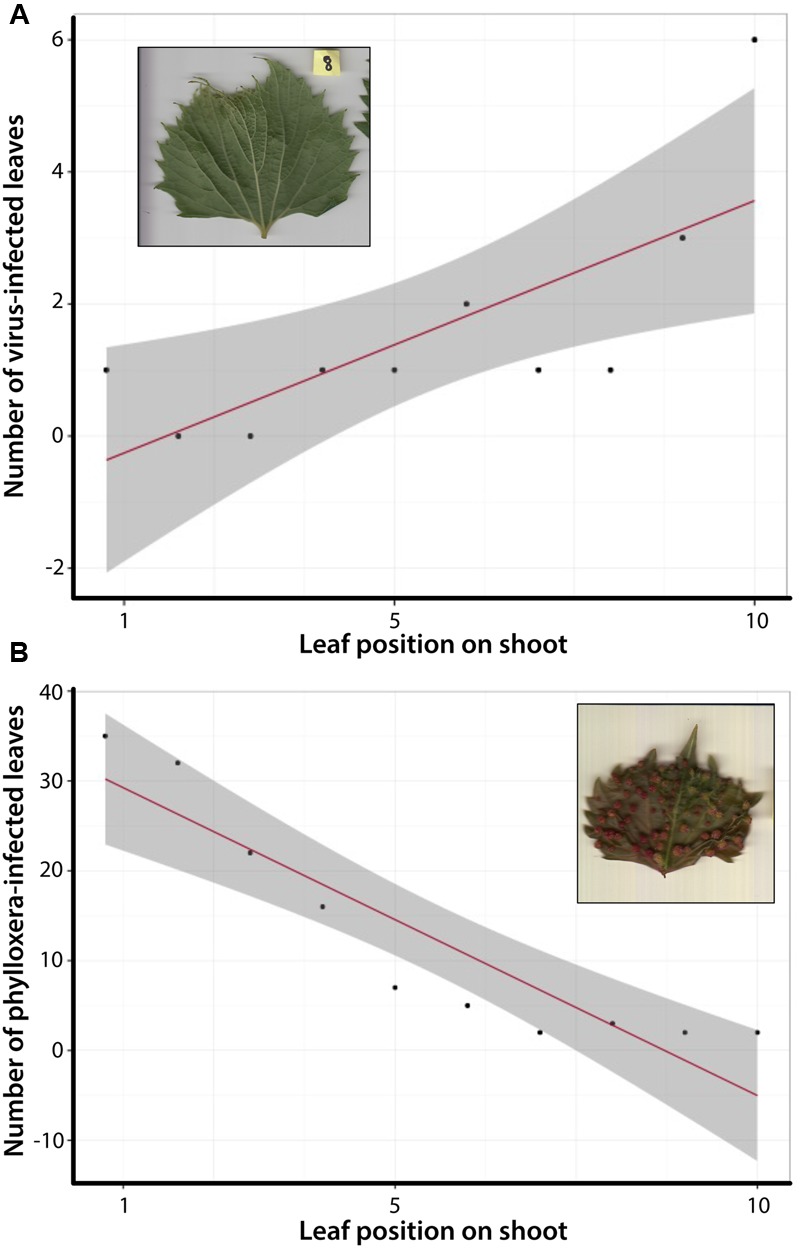
**Correlation tests between shoot position and number of leaves expressing an infected phenotype. (A)** Total number of leaves expressing the GFLV phenotype occurs more often in older, more developed leaves. **(B)** Total number of leaves expressing phylloxera galls is more common in newly developed leaves.

During initial analysis of the total MBG vineyard dataset, we observed several leaves expressing phenotypes congruent with deformities caused by GFLV or galls indicative of phylloxera infestation. Leaves infected with GFLV, a common viral infection among grapevines, exhibit crowding of the major veins into a fan-like shape, with vein tips elongating past the leaf blade at termination (**Figure 4A**). The petiolar sinus, which is concave in healthy leaves of both *V. riparia* and *V. rupestris*, instead becomes convex at the petiolar junction. Phylloxera, also specific to grapevines, is a parasitic insect that forms galls in the leaf and root tissues, causing malformations in the surrounding tissues and structures (**Figure 4B**). Diseased leaf phenotypes resulted in several LDA misidentifications in the total dataset (Supplementary Figure S1). Moreover, we observed that certain genotypes appeared to be more susceptible to pests (e.g., 588347, 588181) or pathogens (e.g., 588439, 588188) while others maintain a healthy phenotype (e.g., 588333, 588160; Supplementary Figure S1). The pattern of incorrect assignment suggests certain genotypes were more susceptible leaf shape modification as a result of pests or pathogens.

We quantified the diseased phenotype further by looking at the relationship between leaf development (position of the leaf along a shoot) and infected leaf phenotypes. For the total dataset of 640 leaves correlation tests demonstrated a significant relationship between older, more developed leaves and the expression of the GFLV phenotype (**Figure 4A**). In contrast, the opposite trend was observed for phylloxera galls, which were more common in younger leaves (**Figure 4B**). These data suggest not only are certain genotypes within a species more susceptible to pathogens than others, but that the expression of unique leaf phenotypes associated with disease varies along the shoot within an individual.

## Discussion

This study advances current understanding of shape differences in the context of species, development, and biotic interaction through the use of clonal replicates of multiple genotypes in a common environment. Digital morphometric techniques offer great utility for future research in *Vitis*, but also serve as an example for other biological systems that seek to make sense of morphological variation.

### Leaf Shape Variation among Species, among Genotypes within Species, and among Clones within Genotypes

Detailed analyses of leaf shape variation have applications in viticulture (Galet, 1979; Chitwood et al., 2014) and also in biodiversity research. Our results indicate that inter- and intra-specific leaf shape variation is discernible in a common garden containing multiple genotypes of *V. riparia* and *V. rupestris*. Distinct clusters of *V. riparia* and *V. rupestris* visualized with GPA (**Figure 2A**) and EFD (**Figure 3B**) confirm existing species distinctions based on morphological and phylogenetic data (Moore, 1991; Ren et al., 2011). Researchers already combine genetic and morphological data to generate strong phylogenetic hypotheses (Cannon and Manos, 2001; Wortley and Scotland, 2006; Fouquet et al., 2012). Increasingly, the utility of digital morphometrics for evolutionary and ecological research is becoming more apparent (Neto et al., 2006; Cope et al., 2012; Wilf et al., 2016), and has promising applications as museum collections become digitized and publicly available. Our analyses also show distinct, averaged leaf shapes among genotypes that are clonally replicated (**Figures 2C, 3A**). In addition, interclonal variation was detected, as LDA identified several individual leaves that were incorrectly assigned (**Table 2**). The ability to quantify discrete phenotypes across multiple levels of organization (within genotype, among genotype, among species) could be serviceable in the identification of adaptive phenotypes linked with genetic or environmental data (e.g., Lande, 2009).

The resolution with which our study identified inter- and intra-specific leaf shape variation is valuable to ecological questions that attempt to discern predictable patterns among complex systems. For example, community interaction or phenotypic plasticity research regularly seeks to make use of functional traits (traits related to increased fitness), which can be related to morphological features. Several studies have identified relationships between leaf shape and altitudinal or temperature gradients using more traditional, length and width leaf measurements or anatomical traits (e.g., *Metrosideros*, Cordell et al., 1998; *Nothofagus*, Hovenden and Vander Schoor, 2004; *Oryza*, Zhou et al., 2013). Applying GPA, LDA, or EFD analysis to morphological data in combination with environmental and genetic data is increasingly feasible as bioinformatic capability increases, thus increasing the potential to uncover acute character linkages between or among species, populations, or individuals. Recently, digital morphometrics was employed to compare *Vitis* leaves from USDA-ARS-GGRU germplasm from two different growing seasons (Chitwood et al., 2016b). In this work, growing season was accurately predicted from leaf shape using LDA. Large-scale digital morphometric datasets have the potential to identify subtle evolutionary and ecological relationships.

### Signatures of Pest and Pathogen Interaction

Digital morphometrics is an effective method for identifying and characterizing biotic stress in plants. Consistent with previous work, we observed that pest and pathogen infestation in grapevines affects specific genotypes more than others (Andret-Link et al., 2004; Granett et al., 2001; Omer et al., 1999b; Supplementary Figure S1). Further, our data suggest there is developmental context to the disease phenotype expressed within a single individual (**Figure 4**). Individuals infected with GFLV expressed the diseased phenotype in more developed leaves; whereas individuals infected with phylloxera expressed the diseased phenotype (leaf galls) in younger leaves. Primary goals of grape breeding include the development of biotic stress resistant scions and rootstocks, and *V. riparia* and *V. rupestris* surveyed here have been used to breed both the rootstock and the scion (Warschefsky et al., 2016). North American grapevines have evolved resistance to native pests and pathogens such as GFLV and phylloxera, but several studies (including ours) suggest variation in resistance response (e.g., Omer et al., 1999a; McKenry et al., 2001). As such it is useful to examine the range of natural variation in native grapevines that could be harnessed to maintain pest and pathogen resistance in grapevines. As researchers continue to investigate these patterns, detailed phenotyping paired with molecular and ecological data could provide deeper insight to these challenges to the grapevine industry.

## Conclusion

In this study, we analyzed leaf shape variation between *V. riparia* and *V. rupestris*, as well as within and among genetically identical individuals of those species. Patterns of morphological differentiation were consistent with species boundaries and displayed genotype-specific signatures. Further, we observed leaf shape variation among clones, some of which was the result of pest and pathogen-induced leaf damage at predictable developmental stages. These data provide a window into how leaf shape varies among species, genotypes, and clones under common conditions, and offers a unique opportunity to look at the effect of abiotic effects on cloned individuals.

## Author Contributions

LK, DC, and AM contributed to the conception of the work and the interpretation of data. LK, MC, CK, and RO contributed to data acquisition, and LK, CC, and DC contributed to analysis. LK drafted the manuscript, and all authors revised several drafts. All authors agree to be accountable for accuracy and integrity of the work.

## Conflict of Interest Statement

The authors declare that the research was conducted in the absence of any commercial or financial relationships that could be construed as a potential conflict of interest.

## Footnotes

1 https://dataverse.harvard.edu/dataverse/VitisLeafVariation

2 https://github.com/llklein/VitisDigitalMorphometricAnalysis

## References

[B1] Abràmoff M. D. Magalhães P. J. Ram S. J. (2004). Image processing with ImageJ. *Biophotonics Int.* 11 36–42.

[B2] Andret-Link P. Laprote C. Valat L. Ritzenthaler C. Demangeat G. Vigne E. (2004). Grapevine fanleaf virus: still a major threat to the grapevine industry. *J. Plant Pathol.* 86 183–195.

[B3] Atlan A. Hornoy B. Delerue F. Gonzalez M. Pierre J. S. Tarayre M. (2015). Phenotypic plasticity in reproductive traits of the perennial shrub Ulex europaeus in response to shading: a multi-year monitoring of cultivated clones. *PLoS ONE* 10:e0137500 10.1371/journal.pone.0137500 PMC4575064 26383627

[B4] Bonhomme V. Picq S. Gaucherel C. Claude J. (2014). Momocs: outline analysis using R. *J. Stat. Softw.* 56 1–24. 10.18637/jss.v056.i13

[B5] Boyce C. K. (2008). The fossil record of plant physiology and development: what leaves can tell us. *Paleontol. Soc. Pap.* 14 133–146.

[B6] Boyd L. A. Ridout C. O’Sullivan D. M. Leach J. E. Leung H. (2013). Plant–pathogen interactions: disease resistance in modern agriculture. *Trends Genet.* 29 233–240. 10.1016/j.tig.2012.10.011 23153595

[B7] Callen S. T. Klein L. L. Miller A. J. (2016). Climatic niche characterization of 13 North American *Vitis* species. *Am. J. Enol. Viticult.* 52 304–309. 10.5344/ajev.2016.15110

[B8] Cannon C. H. Manos P. S. (2001). Combining and comparing morphometric shape descriptors with a molecular phylogeny: the case of fruit type evolution in Bornean *Lithocarpus* (Fagaceae). *Syst. Biol.* 50 860–880. 10.1080/106351501753462849 12116637

[B9] Chitwood D. H. Klein L. L. O’Hanlon R. Chacko S. Greg M. Kitchen C. (2016a). Latent developmental and evolutionary shapes embedded within the grapevine leaf. *New Phytol.* 210 343–355. 10.1111/nph.13754 26580864 PMC5063178

[B10] Chitwood D. H. Ranjan A. Martinez C. C. Headland L. R. Thiem T. Kumar R. (2014). A modern ampelography: a genetic basis for leaf shape and venation patterning in grape. *Plant Physiol.* 164 259–272. 10.1104/pp.113.229708 24285849 PMC3875807

[B11] Chitwood D. H. Rundell S. M. Li D. Y. Woodford Q. L. Yu T. T. Lopez J. R. (2016b). Climate and developmental plasticity: interannual variability in grapevine leaf morphology. *Plant Physiol.* 170 1480–1491. 10.1104/pp.15.01825 26826220 PMC4775139

[B12] Chitwood D. H. Sinha N. R. (2016). Evolutionary and environmental forces sculpting leaf development. *Curr. Biol.* 26 R297–R306. 10.1016/j.cub.2016.02.033 27046820

[B13] Cope J. S. Corney D. Clark J. Y. Remagnino P. Wilkin P. (2012). Plant species identification using digital morphometrics: a review. *Expert Syst. Appl.* 39 7562–7573. 10.1016/j.eswa.2012.01.073

[B14] Cordell S. Goldstein G. Mueller-Dombois D. Webb D. Vitousek P. M. (1998). Physiological and morphological variation in *Metrosideros polymorpha*, a dominant Hawaiian tree species, along an altitudinal gradient: the role of phenotypic plasticity. *Oecologia* 113 188–196. 10.1007/s004420050367 28308196

[B15] De Kroon H. Huber H. Stuefer J. F. Van Groenendael J. M. (2005). A modular concept of phenotypic plasticity in plants. *New Phytol.* 166 73–82. 10.1111/j.1469-8137.2004.01310.x 15760352

[B16] Dkhar J. Pareek A. (2014). What determines a leaf’s shape? *EvoDevo* 5:47 10.1186/2041-9139-5-47 PMC4290414 25584185

[B17] Dryden I. L. (2017). *R Foundation for Statistical Computing. Contributed Package. Version 1.2.0.* Vienna: R Foundation for Statistical Computing.

[B18] Fouquet A. Recoder R. Teixeira M. Jr. Cassimiro J. Amaro R. C. Camacho A. (2012). Molecular phylogeny and morphometric analyses reveal deep divergence between Amazonia and Atlantic Forest species of *Dendrophryniscus*. *Mol. Phylogenet. Evol.* 62 826–838. 10.1016/j.ympev.2011.11.023 22166838

[B19] Galet P. (1979). *A Practical Ampelography.* Ithaca, NY: Cornell University Press.

[B20] Granett J. Walker M. A. Kocsis L. Omer A. D. (2001). Biology and management of grape phylloxera. *Annu. Rev. Entomol.* 46 387–412. 10.1146/annurev.ento.46.1.387 11112174

[B21] Hovenden M. J. Vander Schoor J. K. (2004). Nature vs nurture in the leaf morphology of Southern beech, *Nothofagus cunninghamii* (Nothofagaceae). *New Phytol.* 161 585–594. 10.1046/j.1469-8137.2003.00931.x 33873506

[B22] Ichihashi Y. Aguilar-Martínez J. A. Farhi M. Chitwood D. H. Kumar R. Millon L. V. (2014). Evolutionary developmental transcriptomics reveals a gene network module regulating interspecific diversity in plant leaf shape. *Proc. Natl. Acad. Sci. U.S.A.* 111 E2616–E2621. 10.1073/pnas.1402835111 24927584 PMC4078850

[B23] Iwata H. Ukai Y. (2002). SHAPE: a computer program package for quantitative evaluation of biological shapes based on elliptic Fourier descriptors. *J. Hered.* 93 384–385. 10.1093/jhered/93.5.384 12547931

[B24] Kadioglu A. Terzi R. Saruhan N. Saglam A. (2012). Current advances in the investigation of leaf rolling caused by biotic and abiotic stress factors. *Plant Sci.* 182 42–48. 10.1016/j.plantsci.2011.01.013 22118614

[B25] Kaplan D. R. (2001). The science of plant morphology: definition, history, and role in modern biology. *Am. J. Bot.* 88 1711–1741. 10.2307/3558347 21669604

[B26] Lande R. (2009). Adaptation to an extraordinary environment by evolution of phenotypic plasticity and genetic assimilation. *J. Evol. Biol.* 22 1435–1446. 10.1111/j.1420-9101.2009.01754.x 19467134

[B27] Little S. A. Kembel S. W. Wilf P. (2010). Paleotemperature proxies from leaf fossils reinterpreted in light of evolutionary history. *PLoS ONE* 5:e15161 10.1371/journal.pone.0015161 PMC3008682 21203554

[B28] McKenry M. V. Kretsch J. O. Anwar S. A. (2001). Interactions of selected rootstocks with ectoparasitic nematodes. *Am. J. Enol. Vitic.* 52 304–309.

[B29] Miller A. J. Matasci N. Schwaninger H. Aradhya M. K. Prins B. Zhong G. Y. (2013). Vitis phylogenomics: hybridization intensities from a SNP array outperform genotype calls. *PLoS ONE* 8:e78680 10.1371/journal.pone.0078680 PMC3827278 24236035

[B30] Moore M. O. (1991). Classification and systematics of eastern North American *Vitis* L. (Vitaceae) north of Mexico. *SIDA Contrib. Bot.* 14 339–367.

[B31] Moore M. O. Wen J. (2016). “Vitaceae,” in *Flora of North America North of Mexico* Vol. 12 ed. Flora of North America Editorial Committee (New York, NY: Oxford University Press).

[B32] Nabity P. D. Haus M. J. Berenbaum M. R. DeLucia E. H. (2013). Leaf-galling phylloxera on grapes reprograms host metabolism and morphology. *Proc. Natl. Acad. Sci. U.S.A.* 110 16663–16668. 10.1073/pnas.1220219110 24067657 PMC3799386

[B33] Neto J. C. Meyer G. E. Jones D. D. Samal A. K. (2006). Plant species identification using Elliptic Fourier leaf shape analysis. *Comput. Electron. Agric.* 50 121–134. 10.1016/j.compag.2005.09.004

[B34] Nicotra A. B. Leigh A. Boyce C. K. Jones C. S. Niklas K. J. Royer D. L. (2011). The evolution and functional significance of leaf shape in the angiosperms. *Funct. Plant Biol.* 38 535–552. 10.1098/rspb.2012.1110 32480907

[B35] Omer A. D. Granett J. Kocsis L. Downie D. A. (1999a). Preference and performance responses of California grape phylloxera to different *Vitis* rootstocks. *J. Appl. Entomol* 123 341–346. 10.1046/j.1439-0418.1999.00394.x

[B36] Omer A. D. Granett J. Wakeman R. J. (1999b). Pathogenicity of *Fusarium oxysporum* on different *Vitis* rootstocks. *J. Phytopathol.* 147 433–436. 10.1111/j.1439-0434.1999.tb03846.x

[B37] Peppe D. J. Royer D. L. Cariglino B. Oliver S. Y. Newman S. Leight E. (2011). Sensitivity of leaf size and shape to climate: global patterns and paleoclimatic applications. *New Phytol.* 190 724–739. 10.1111/j.1469-8137.2010.03615.x 21294735

[B38] R Core Team (2016). *R: A Language and Environment for Statistical Computing.* Vienna: R Foundation for Statistical Computing.

[B39] Ren H. Lu L. M. Soejima A. Luke Q. Zhang D. X. Chen Z. D. (2011). Phylogenetic analysis of the grape family (Vitaceae) based on the noncoding plastid trnC-petN, trnH-psbA, and trnL-F sequences. *Taxon* 60 629–637.

[B40] Rendu V. (1854). *Ampélographie Française: Description des Principaux Cépages, des Procédés de Culture et de Vinification Usités dans les Meilleurs Crus de France.* Paris: V. Bouchard-Huzard.

[B41] Royer D. L. Meyerson L. A. Robertson K. M. Adams J. M. (2009). Phenotypic plasticity of leaf shape along a temperature gradient in *Acer rubrum*. *PLoS ONE* 4:e7653 10.1371/journal.pone.0007653 PMC2764093 19893620

[B42] Venables W. N. Ripley B. D. (2002). *Modern Applied Statistics with S* 4th Edn. New York, NY: Springer 10.1007/978-0-387-21706-2

[B43] Viscosi V. Cardini A. (2011). Leaf morphology, taxonomy and geometric morphometrics: a simplified protocol for beginners. *PLoS ONE* 6:e25630 10.1371/journal.pone.0025630 PMC3184990 21991324

[B44] Wan Y. Schwaninger H. R. Baldo A. M. Labate J. A. Zhong G.-Y. Simon C. J. (2013). A phylogenetic analysis of the grape genus (*Vitis* L.) reveals broad reticulation and concurrent diversification during neogene and quaternary climate change. *BMC Evol. Biol.* 13:141 10.1186/1471-2148-13-141 PMC3750556 23826735

[B45] Warschefsky E. J. Klein L. L. Frank M. H. Chitwood D. H. Londo J. P. von Wettberg E. J. B. (2016). Rootstocks: diversity, domestication, and impacts on shoot phenotypes. *Trends Plant Sci.* 21 418–437. 10.1016/j.tplants.2015.11.008 26698413

[B46] Wickham H. (2009). *Ggplot2: Elegant Graphics for Data Analysis.* Berlin: Springer Science & Business Media 10.1007/978-0-387-98141-3

[B47] Wilf P. Zhang S. Chikkerur S. Little S. A. Wing S. L. Serre T. (2016). Computer vision cracks the leaf code. *Proc. Natl. Acad. Sci. U.S.A.* 113 3305–3310. 10.6084/m9.figshare.1521157 26951664 PMC4812720

[B48] Wolf S. D. Silk W. K. Plant R. E. (1986). Quantitative patterns of leaf expansion: comparison of normal and malformed leaf growth in *Vitis vinifera* cv. Ruby Red. *Am. J. Bot.* 73 832–846. 10.2307/2444294

[B49] Wortley A. Scotland R. (2006). The effect of combining molecular and morphological data in published phylogenetic analyses. *Syst. Biol.* 55 677–685. 10.1080/10635150600899798 16969943

[B50] Zhou W. Wang Z. Davy A. J. Liu G. (2013). Geographic variation and local adaptation in *Oryza rufipogon* across its climatic range in China. *J. Ecol.* 101 1498–1508. 10.1111/1365-2745.12143

