# Supplementary figures and images for: Digital Morphometrics of Two North American Grapevines (Vitis: Vitaceae) Quantifies Leaf Variation between Species, within Species, and among Individuals
Source: Front Plant Sci. 2017 Mar 17;8:373. doi: 10.3389/fpls.2017.00373 (PMC5355467; doi:10.3389/fpls.2017.00373)

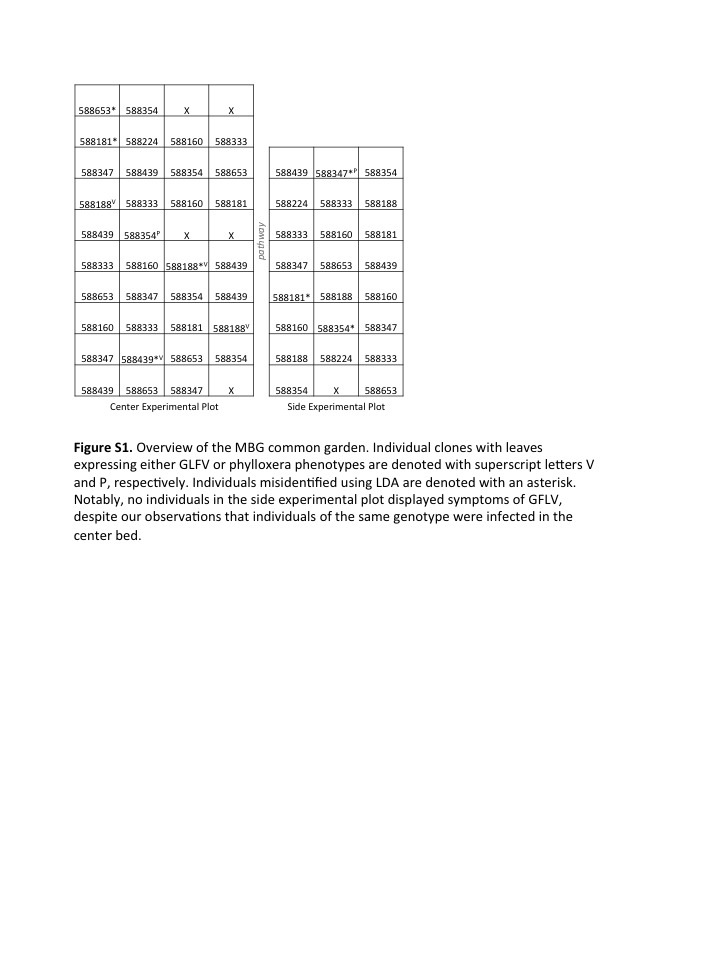

Supplement: Supplementary file 2 [file Image_1.TIFF]
